# Evolution of Methods for the Quantitative Assessment of Inbreeding in Livestock

**DOI:** 10.3390/biology15070530

**Published:** 2026-03-26

**Authors:** Lyubov Getmantseva, Siroj Bakoev, Maria Kolosova, Alexandr Usatov, Kharon Amerkhanov, Olga Lukonina

**Affiliations:** 1 All Russian Research Institute of Animal Breeding, Lesnye Polyany 141212, Russia; ilonaluba@mail.ru (L.G.); lukoninaon@mail.ru (O.L.); 2 Institute of Animal Science and Biology, Russian State Agrarian University-Moscow Agricultural Academ Named After K.A. Timiryazev, Moscow 127434, Russia; h.amerhanov@rgau-msha.ru; 3 Academy of Biology and Biotechnology Named After D.I. Ivanovsky, Southern Federal University, Rostov-on-Don 344006, Russia; usatova@mail.ru

**Keywords:** livestock, inbreeding, autozygosity, ROH, HBD, IBD, pedigree

## Abstract

Inbreeding is a quantitative measure of autozygosity that underlies the assessment of genetic risks and the management of genetic progress in livestock populations. The development of methods for its estimation reflects a transition from probabilistic pedigree-based models to the direct analysis of genome structure. This review systematizes the evolution of approaches to inbreeding assessment—from the classical inbreeding coefficient F based on identity by descent (IBD) to marker-based, segment-based runs of homozygosity (ROH) and probabilistic homozygous-by-descent (HBD) models. It is shown that the coefficients F_ped, F_GRM, F_ROH, and F_HBD capture related but distinct aspects of autozygosity and are therefore not fully interchangeable. Particular attention is paid to the transition from integral indicators to spatially and temporally stratified analyses of autozygosity, enabling the differentiation between ancient and recent inbreeding. Methodological assumptions, limitations, and the sensitivity of various approaches to marker density, detection parameters, and population demographic structure are discussed. A comparative analysis of methods for calculating F_ROH and segment-based autozygosity is presented. The necessity of a comprehensive assessment of inbreeding and the standardization of analytical protocols for its application in modern breeding programs is substantiated.

## Simple Summary

Modern genomic methods enable precise assessment of inbreeding at the DNA segment level in livestock populations. This review traces the evolution of approaches from classical pedigree-based F coefficients to marker-based (GRM), segmental (ROH analysis), and probabilistic HBD models (PLINK, DetectRUN, RZooRoH). The authors emphasize differences between F_ped, F_GRM, F_ROH, and F_HBD, as well as the ability of ROH/HBD methods to distinguish recent inbreeding (long segments causing depression) from ancient inbreeding (short segments). Understanding these methods is essential for standardizing protocols and effective genetic diversity management in breeding programs.

## 1. Introduction

Inbreeding (i.e., homozygosity by descent) in animal breeding has very deep historical roots. Initially, it is associated with domestication (on average 10–15 thousand years ago for different species); subsequently, purposeful selection, breed formation, intensive selection for productivity, and the globalization of genetics (the worldwide dissemination of a few highly productive lines) have led to the formation of extended regions of homozygosity and a reduction in genetic diversity [1]. The balance between genetic improvement (high productivity) and the maintenance of genetic diversity remains a key challenge in modern animal production.

In closed populations, there is a high risk of increased inbreeding pressure associated with the founder effect, as such populations are formed from a limited number of elite animals. As a result, the genetic diversity of the population is determined by a small number of original founders. Consequently, breeders have faced the dilemma that, on the one hand, inbreeding is useful for fixing desirable traits, but on the other hand, it leads to reduced fitness. Outbreeding, in contrast, increases heterozygosity and resilience, but may “dilute” valuable traits.

Effective management of the breeding process and reduction in biological risks required the development of quantitative methods for assessing relatedness, and the creation of approaches for measuring inbreeding became a crucial milestone that enabled the transition from practical experience to scientifically grounded breeding strategies.

The aim of this review is to trace the evolution of scientific concepts of inbreeding and its measurement—from the classical probabilistic concept of autozygosity in pedigree-based models to modern genomic approaches based on marker data and the segmental structure of the genome. Particular attention is given to how advances in molecular technologies have expanded the possibilities for quantitative assessment of inbreeding, enabling the transition from its expected probabilistic characterization to the analysis of the realized segmental structure of the genome and the age stratification of autozygosity. In this context, pedigree-based theory is viewed as the conceptual foundation of modern genomic approaches rather than as an alternative to ROH- and HBD-based methods.

### 1.1. Methodology of Literature Selection

The literature included in this review was identified through a structured search in major scientific databases, including Web of Science, Scopus, and PubMed. The search was performed using combinations of keywords such as “inbreeding coefficient”, “autozygosity”, “ROH”, “HBD”, “genomic inbreeding”, and “livestock”, combined using Boolean operators (AND, OR). In addition, relevant regional and non-English literature was considered where appropriate.

The inclusion criteria focused on (i) methodological relevance to inbreeding estimation (pedigree-, GRM-, ROH-, and HBD-based approaches), (ii) application to livestock populations, and (iii) publication in peer-reviewed journals.

Studies were excluded if they were purely theoretical without validation or not directly related to inbreeding assessment.

### 1.2. Evolution of the Concept of Inbreeding and Autozygosity in Population Genetics

The inbreeding coefficient (F) is a key parameter in population genetics that reflects the probability of homozygosity of alleles in an individual due to related matings. The concept of the inbreeding coefficient (F) and the method for its calculation were first proposed in the 1920s by the American geneticist and one of the founders of population genetics, Sewall Wright [2]. Sewall Wright developed a method for calculating the inbreeding coefficient (F), defined as the probability that two alleles at a locus are identical by descent.

The method is based on the analysis of the pedigree structure of a population and involves accounting for all common ancestors of an individual’s parents; the lengths of inheritance paths from each ancestor to the sire and dam; as well as the inbreeding status of the ancestors themselves [3].

The development of inbreeding research after the work of Sewall Wright proceeded in several directions, ranging from the refinement of mathematical models to applications in genetics, breeding, and conservation biology. The French mathematician and geneticist Gustave Malécot formalized the theory of inbreeding through probabilistic models, linking them to genetic drift and population size [4]. He provided a formal probabilistic interpretation of relatedness/coancestry and connected it with drift and effective population size. His work laid the foundation for modern population-genetic models.

In the 1940s, Charles Cotterman developed a system of identity coefficients describing the probabilities of alleles being identical by descent, thereby establishing the basis for the quantitative analysis of genetic relatedness [5,6]. In modern terminology, this corresponds to identity by descent (IBD) and identity by state (IBS) [7].

Identity by descent (IBD) means that matching alleles in two individuals originate from the same ancestral allele [5,8]. Such identity reflects true genealogical relatedness and underlies the concept of inbreeding as homozygosity by descent. In contrast, identity by state (IBS) refers only to the coincidence of allelic states and is not necessarily associated with a common ancestor; therefore, by itself, it is not an indicator of relatedness.

Cotterman demonstrated that, for the assessment of inbreeding, it is essential to consider IBD specifically, since only identity by descent reflects the degree of related mating and the accumulation of autozygosity in a population. These principles became the theoretical foundation of modern methods for the analysis of pedigree and genomic data [9].

Thus, the concept of IBD formulated in Cotterman’s work bridges the classical theory of pedigree-based inbreeding and contemporary genomic approaches to the assessment of relatedness and population structure. The coefficient of relationship is a fundamental parameter linking genetics and breeding. It can be calculated using both pedigree and genomic data [10].

The inbreeding coefficient (F) was first integrated into breeding programs and its impact on productivity and health in livestock populations was quantitatively evaluated by the American geneticist Jay L. Lush [11]. Jay Lush is considered the “father of scientific animal breeding” for his systematic approach to livestock improvement. He demonstrated that the accumulation of inbreeding leads to inbreeding depression, reduced fertility, decreased offspring viability, and lower adaptability. His work laid the foundations of controlled inbreeding, in which close matings are used to fix desirable traits but are strictly regulated. Lush also developed principles for estimating breeding value that account not only for phenotype but also for relatedness among animals. These methods are still applied in modern animal improvement programs, now in combination with genomic selection [12,13].

The work of Jay L. Lush on controlled inbreeding and breeding value estimation was reflected and further developed in the studies of Russian and Soviet scientists. One of the founders of Russian animal science, P.N. Kuleshov, identified inbreeding as a key mechanism for hereditary consolidation. Yet, he cautioned that its success depends on a multi-faceted approach involving strict selection and improved environmental conditions [14].

An outstanding example of the practical application of inbreeding in combination with other breeding methods is found in the work of Academician M.F. Ivanov [15]. Relying on moderate inbreeding, the use of outstanding ancestors, and strict selection, he developed world-renowned breeds (the Askanian fine-wool sheep and the Ukrainian Steppe White pig), and his breed-formation methodology became a classic of breeding science [16].

The British scientist Alan Robertson made a fundamental contribution to understanding the dynamics of inbreeding under artificial selection. In a series of studies published in the 1950s–1960s, he demonstrated that intensive selection leads to a reduction in effective population size (Ne) due to the unequal contribution of individual families to the next generation [17]. This, in turn, accelerates the accumulation of inbreeding compared with expectations for an idealized random-mating population.

Robertson showed that the rate of increase in the inbreeding coefficient depends on selection intensity, trait heritability, and population structure [18]. Thus, in his models, inbreeding appears as an inevitable consequence of selection: on the one hand, it promotes the fixation of favorable alleles and enhances selection response; on the other hand, it reduces genetic variability and may limit long-term genetic progress.

These theoretical principles formed the basis for the subsequent development of methods to manage the genetic contributions of breeding animals and to limit the rate of inbreeding increase. This later led to the emergence of the concept of optimal allocation of matings and the controlled use of lines in breeding programs [19].

### 1.3. Estimation of Inbreeding Based on Pedigree Data and Its Limitations

The estimation of inbreeding based on pedigree data is historically the first and one of the most thoroughly studied methods. Among its advantages is that it performs well when pedigrees are complete and accurate and does not require genetic data. However, its limitations are related to the need for comprehensive and reliable pedigree information extending several generations back, as well as the assumption of no selection and random mating. In addition, this method does not account for possible errors in pedigree records, fails to detect distant inbreeding, and does not consider the stochastic processes of Mendelian segregation (recombination) [20].

When calculating the inbreeding coefficient based on pedigree data, several key assumptions are made regarding the relationships among the founders (base animals) and the absence of selection; that is, the transmission of genetic material from parents to offspring is assumed to occur purely at random.

The assumption regarding pedigree founders is based on the premise that all founders (animals for which no ancestral information is available) are unrelated to each other; that is, all founders are considered genetically equally distant and to have no common ancestors. This simplification makes it possible to calculate inbreeding in subsequent generations while excluding potential genetic similarities among founders that may have existed in reality [21]. However, if the founders are in fact related, the classical pedigree-based calculation of inbreeding will systematically underestimate the true level of inbreeding. In some populations, especially small or isolated ones, founders almost always share some degree of relatedness.

The assumption of no selection is based on the idea that the transmission of genetic material from parents to offspring occurs purely at random, and that genes are inherited independently and without any selective influence. However, when animals with superior performance are selected, desirable alleles are subject to selection; therefore, the transmission of genetic material is not random [22]. Moreover, selection acts not on individual genes but on genomic regions (linked segments), which may subsequently be reshaped by recombination. As a result, the actual genetic diversity may be substantially lower, and the true level of inbreeding higher, than that estimated from pedigree data alone.

In populations that have developed in relative isolation over long periods of time, genetic diversity gradually declines due to the limited number of individuals and the inevitable occurrence of related matings [23]. In such populations, the pedigree-based inbreeding coefficient typically underestimates the true degree of genetic relatedness, as it does not account for distant relationships that existed prior to the onset of recorded breeding.

In addition, it is important to consider the baseline (background) level of inbreeding present in domestic animal populations, which cannot be captured when pedigrees are incomplete. Beyond the classical pedigree-based inbreeding coefficient, several extended pedigree-derived measures have been proposed to partition inbreeding into ancestral and new components. The ancestral inbreeding coefficient introduced by Ballou reflects the probability that alleles have been autozygous in previous generations, whereas the framework of Kalinowski distinguishes between ancestral and new inbreeding, providing a pedigree-based approximation of older versus more recent autozygosity [24,25]. These coefficients are particularly relevant in studies of purging and inbreeding depression. However, like other pedigree-based measures, they depend on pedigree completeness and reflect expected rather than realized genomic inheritance.

### 1.4. Marker-Based and Matrix-Based Genomic Estimates of Inbreeding

The development of molecular genetic technologies and the widespread adoption of SNP genotyping have enabled a transition from estimating expected inbreeding to assessing its realized level using genomic data. Unlike pedigree-based approaches, genomic methods do not depend on the completeness or depth of genealogical information and allow the quantitative evaluation of autozygosity directly at the DNA level. This has led to the emergence of a new class of inbreeding estimation methods based on the analysis of marker homozygosity and genomic relationships between individuals [1].

At the early stage of genomic approaches, the inbreeding coefficient was mainly estimated based on the deviation of the observed level of homozygosity from that expected under Hardy–Weinberg equilibrium. This estimate reflects the realized level of genomic homozygosity; however, it characterizes autozygosity only in an aggregated form and does not provide information about the spatial distribution of homozygous segments across the genome.

Further development of genomic methods led to the use of genomic relationship matrices (genomic relationship matrix, GRM), which quantitatively describe the degree of genetic similarity between individuals based on marker data. Within the most widely used approach proposed by VanRaden, the GRM is constructed using normalized SNP genotypes while accounting for allele frequencies in the population [26]. In this case, the inbreeding coefficient of an individual can be approximated by the diagonal element of the genomic relationship matrix. Such an estimate is interpreted as a measure of realized inbreeding in the space of genomic relationships and is widely applied in genomic selection models, such as GBLUP (genomic best linear unbiased prediction), which is based on the use of the genomic relationship matrix, and ssGBLUP (single-step genomic best linear unbiased prediction), which integrates genomic and pedigree data into a unified model for breeding value estimation. However, the value of *Fi* depends on the method used to construct the GRM and on the chosen normalization scale, which complicates the direct comparison of results across different studies [27,28].

These methods made it possible to estimate realized inbreeding and are widely applied in population genetics and genomic selection. However, they characterize only the overall level of autozygosity and do not allow the contributions of recent and ancient common ancestors to be distinguished. The limitations of these approaches stimulated the development of inbreeding estimation methods based on the analysis of runs of homozygosity (ROH-based measures of inbreeding), in which autozygosity is considered not as an aggregated value but as a spatially distributed property of the genome.

### 1.5. Segment-Based Methods for Estimating Inbreeding Using ROH

An important stage in the development of genomic methods for inbreeding estimation was the use of the analysis of extended homozygous genomic regions (runs of homozygosity, ROH), which made it possible to move from integral estimates of autozygosity to its spatially and temporally structured description [29]. ROH are long continuous regions of homozygosity that arise when an individual inherits two copies of the same haplotype from common ancestors [30]. Since the 2010s, ROH analysis has been widely applied in studies of domestic animals and has become one of the standard tools for investigating inbreeding and signatures of selection [31].

Methods for estimating inbreeding based on ROH analysis involve the identification of continuous homozygous segments within which both alleles originate from a common ancestor. Based on these extended homozygous regions, the inbreeding coefficient.

F_ROH is calculated as the proportion of the genome covered by ROH segments. Unlike pedigree-based estimates of inbreeding, this indicator does not require information about the ancestry of individuals and reflects the realized level of autozygosity directly at the genomic level. A major advantage of ROH analysis is the ability to account not only for the overall level of inbreeding but also for its temporal structure [32].

Nevertheless, the practical application of ROH analysis imposes increased requirements on the quality and density of genomic data and is sensitive to the parameters used for detecting homozygous segments. The need for a more rigorous treatment of the uncertainty in the origin of such segments has led to the development of probabilistic models in which the genome is described as a mosaic of regions that are homozygous by descent (HBD), distributed across classes corresponding to different generations of common ancestors [33]. These models allow a more formalized approach to the analysis of the temporal structure of inbreeding and the genetic structure of populations.

The length of ROH segments is closely related to the age of inbreeding. Long segments usually indicate recent common ancestors, whereas shorter segments reflect more ancient events associated with founder effects, demographic bottlenecks, or long-term selection [34]. Thus, ROH analysis makes it possible to distinguish between the contributions of recent and ancient inbreeding, which reflect different demographic and selective processes, and is therefore widely used to reconstruct the demographic history of populations.

The spatial distribution of ROH along chromosomes allows the analysis of autozygosity at a local level and the comparison of homozygous segments with specific genomic regions, including quantitative trait loci and genes associated with economically important and adaptive traits. This makes ROH analysis an important tool for more accurate estimation of inbreeding and its application in breeding programs, as well as for studying the functional consequences of autozygosity through the analysis of genomic regions enriched with genes and loci associated with productive and adaptive traits [35,36].

### 1.6. Probabilistic HBD Models and Age Stratification of Inbreeding

The estimation of inbreeding based on runs of homozygosity (ROH) and homozygous-by-descent (HBD) segments has undergone substantial development over the past two decades, evolving from simple models to complex recursive frameworks capable of accounting for the age structure of inbreeding. The first formalization of HBD segments as alternating regions within the genome was proposed by Leutenegger et al. [37]. Their model (implemented in the Festim package [38] describes the genome as a sequence of two hidden states: HBD and non-HBD. Transitions between these states are controlled by a hidden Markov model (HMM), and the lengths of HBD segments follow an exponential distribution with a parameter related to the number of generations to the common ancestor. The time spent in the HBD state allows the estimation of the individual inbreeding coefficient. This model is referred to as the “1R” model, reflecting the presence of a single HBD class.

As genomic datasets accumulated, it became evident that grouping all HBD segments into a single category does not adequately reflect the differences between ancient and recent inbreeding. Researchers began classifying ROH by length, considering short segments as the result of ancient inbreeding and long segments as indicators of recent inbreeding [39]. This stimulated the development of models that account for multiple age classes of HBD.

Druet and Gautier proposed an extension of the basic HMM in which the HBD state is divided into K classes with different expected segment lengths (1/Rk cM), corresponding to different numbers of generations to a common ancestor [40]. This model makes it possible to estimate total inbreeding and to partition it across ancestral layers, while also providing local probabilities that a genomic region belongs to each HBD class. The model is implemented in the widely used RZooRoH package, which is applied for HBD analysis using both SNP panel and sequencing data [36,40].

Despite these advances, a model with a fixed “non-HBD” state may incorrectly interpret very ancient HBD segments as completely unrelated to inbreeding. This issue is particularly pronounced in populations with high levels of inbreeding, where confusion between truly non-HBD segments and ancient HBD segments leads to distortions in the probability distribution.

To overcome these limitations, a recursive HMM based on the concept of nested ancestral layers was proposed [36]. In this model, each level describes a distribution between HBD and non-HBD states, but the “non-HBD” component of the previous level becomes a mixture of HBD/non-HBD states at the next layer. Thus, the genome is represented as a nested system in which each HBD class corresponds to a specific ancestral epoch. This framework allows a more accurate differentiation between ancient and recent HBD segments and prevents the inflation of probabilities in the most distant classes. In practice, 6–12 layers are typically used, often with parameters R = {2, 4, 8, …, 512}.

The development of nested HBD-class models has made it possible to overcome the key limitations of earlier multiclass approaches and to move toward a more realistic representation of autozygosity within the genome. Unlike models with a fixed non-HBD state, the recursive approach provides a continuous representation of inbreeding along a temporal scale, which substantially improves the accuracy of HBD-segment dating and the correct estimation of the contributions of ancient and recent common ancestors.

By accounting more rigorously for the age structure of inbreeding, nested HBD models allow not only the estimation of integral inbreeding measures but also the analysis of its structure with respect to the time of origin of common ancestors. This is particularly important for interpreting genetic load and assessing the consequences of inbreeding for productive and adaptive traits [41].

Overall, the development of ROH-and HBD-based approaches has led to the emergence of several practical algorithms and software implementations that differ in their assumptions, detection parameters, and interpretation of results. This makes their comparative analysis particularly relevant when estimating the inbreeding coefficient F_ROH.

### 1.7. Methods for Calculating F_ROH and Segment-Based Autozygosity

The methods implemented in software designed to detect ROH segments can generally be classified as observational approaches, model-based approaches, and methods aimed at detecting short homozygous sequences. Despite this conditional classification, most of these programs are capable of addressing common tasks related to the detection and identification of ROH.

PLINK is one of the most widely used software tools for genetic data analysis, including the detection of runs of homozygosity (ROH) [42]. It employs a sliding-window approach to identify consecutive homozygous SNPs. ROH are defined based on the continuity of homozygous SNP sequences while allowing a limited number of missing or heterozygous markers.

DetectRUNS is a specialized R package for detecting and analyzing homozygous regions. It is implemented in the R programming language and provides extensive visualization capabilities [43]. The package supports several algorithms for ROH detection: the sliding-window method (similar to PLINK), the consecutive homozygous SNP method, and a combined approach. It also offers detailed visualization of ROH distribution across chromosomes and by segment length.

RZooRoH represents an advanced approach for ROH detection based on hidden Markov models (HMM) [36]. It uses HMM to model the genome as a mosaic of segments of common ancestry. The method classifies ROH into homozygous-by-descent (HBD) classes according to parameters related to the time since inheritance from common ancestors and explicitly accounts for demographic history and genetic processes.

Each of these methods is freely available and has its own advantages and limitations (Table 1). The advantages of PLINK include computational efficiency, high processing speed even for large datasets, low memory requirements, a wide range of adjustable parameters that allow optimization for different datasets, and extensive representation in the scientific literature.

The disadvantages of PLINK include high sensitivity to parameter selection, where the results may vary substantially depending on the chosen threshold values. Parameter optimization must often be performed separately for each dataset, as universal parameter settings are lacking. In addition, PLINK has limited ability to model complex demographic histories, does not account for demographic changes, and does not estimate the origin or age of ROH segments. The method may also produce type I and type II errors (false-positive and false-negative ROH calls).

The advantages of DetectRUNS include extensive visualization capabilities and detailed classification of ROH by length, allowing the distinction between ancient and recent inbreeding. The package also includes built-in tools for the analysis of ROH results and additional statistical instruments for downstream analysis, as well as integration with the R ecosystem.

The limitations of DetectRUNS include higher computational requirements and greater resource consumption compared with PLINK when working with large datasets. The software can also be slower when processing high-density SNP data and requires familiarity with R programming for effective use. Furthermore, despite improved visualization capabilities, the underlying ROH detection approach remains largely deterministic.

The advantages of RZooRoH include a robust probabilistic framework based on hidden Markov models (HMM). The method explicitly models uncertainty in the identification of ROH segments and is more tolerant to genotyping errors and missing data. It is less sensitive to the choice of specific threshold parameters and accounts for demographic history. In addition, it models multiple HBD classes corresponding to different temporal scales of inbreeding, allowing both recent and ancient inbreeding to be analyzed without relying solely on ROH length. This approach provides high accuracy, reduces the risk of misclassifying genomic segments as ROH, and shows better agreement with true inbreeding levels in simulation studies.

The disadvantages include high computational complexity and greater demands on computational resources compared with PLINK. Difficulties may arise when working with very large datasets, and computation time can be substantial, especially when fine-tuning model parameters. The method also requires specialized expertise: the complexity of the model demands an understanding of hidden Markov models, interpretation of the results requires advanced statistical knowledge, and model parameterization can be challenging. In particular, it is necessary to configure HMM parameters and determine the appropriate number and classes of HBD states, which involves complex optimization procedures.

The choice of method for calculating FROH depends on the specific research objectives, available resources, and practical needs of the livestock sector. PLINK remains a standard, reliable, and computationally efficient tool for large-scale screening studies, although it is sensitive to parameter selection. DetectRUNS provides enhanced visualization and classification of ROH segments, which is particularly useful for detailed analyses. RZooRoH, in turn, represents the most advanced approach, capable of accounting for demographic history and providing probabilistic classification of ROH segments. Despite its higher computational requirements and methodological complexity, it is often preferred for in-depth studies and for critical breeding decisions, such as the selection of elite breeding animals, optimization of mating schemes, and the implementation of programs aimed at improving efficiency while conserving genetic resources.

## 2. Discussion

The evolution of methods for estimating inbreeding reflects a deeper shift in the understanding of genetic autozygosity—from calculating the expected probability of identity by descent to directly measuring realized homozygosity at the genomic level. In this context, it is important to emphasize that the coefficients F_ped, F_GRM, F_ROH, and F_HBD are not fully interchangeable (Table 2), as they capture related but distinct aspects of genetic information organization.

The pedigree-based inbreeding coefficient (F_ped) represents an estimate of expected autozygosity assuming that the ancestral structure is correctly specified. However, in real populations, pedigree completeness, recording depth, and the assumption of unrelated founders are often violated, which leads to systematic discrepancies between expected and realized levels of homozygosity. Comparative studies in pigs, sheep, and cattle show that genomic metrics (F_ROH, F_HBD) reveal more pronounced and biologically meaningful effects of inbreeding depression than F_ped [44,45,46]. This indicates the limitations of interpreting F_ped as a universal indicator of genetic risk.

Marker-based estimates derived from the genomic relationship matrix (F_GRM) represent the next stage in the development of inbreeding assessment; however, their magnitude strongly depends on the reference allele frequency base and the normalization procedure used [33]. Consequently, the absolute values of F_GRM are not invariant across populations or temporal datasets. Moreover, GRM-based inbreeding reflects aggregated genomic homozygosity and does not allow the contribution of recent and ancient common ancestors to be distinguished, which limits its biological interpretability.

Methods based on the analysis of runs of homozygosity (ROH) fundamentally changed the structure of inbreeding analysis by enabling a transition from an integral estimate to a spatially distributed characterization of the genome. However, ROH are not an “objective” quantity outside the context of detection parameters. Marker density, genome assembly quality, minimum segment length, and allowances for heterozygosity substantially influence the number and distribution of detected ROH [12]. Consequently, ROH analysis requires standardization and transparent reporting; otherwise, interpopulation comparisons become methodologically unreliable.

A key conceptual shift in recent years is the recognition that the biological consequences of inbreeding are determined not so much by its overall level as by the age structure of autozygosity. Accumulating evidence demonstrates that long ROH, reflecting recent inbreeding, are statistically associated with reduced fitness traits-including survival, fertility, and productivity [47,48,49]. In contrast, short ROH corresponding to ancient autozygosity are often neutral or exhibit weaker effects, which is consistent with the hypothesis of partial purging of deleterious recessive alleles in previous generations. Thus, inbreeding should be considered as a temporally stratified phenomenon rather than as a homogeneous quantity.

An additional complicating factor is the heterogeneous local architecture of inbreeding depression. Several studies indicate that adverse effects are not driven by a uniform increase in F_ROH, but rather by homozygosity in a limited number of genomic segments with large effects [50,51,52]. In some populations, inbreeding depression appears to be predominantly “genome-wide distributed,” whereas in others it is “regionally concentrated.” These findings point to a population-specific architecture of inbreeding load and highlight the need for combined analyses conducted both at the whole-genome level and at the level of individual genomic segments.

HBD models, particularly nested-layer schemes, provide the most formalized representation of the age structure of autozygosity [36]. Their advantage lies not only in the probabilistic classification of segments but also in the ability to decompose total inbreeding into temporal components, making it possible to directly test hypotheses about the different biological roles of ancient and recent homozygosity. In this way, HBD-based approaches elevate the analysis of inbreeding to the level of dynamic modeling of the demographic and selective history of a population.

From a practical perspective, this implies that the management of inbreeding in modern breeding programs should rely not on a single integral coefficient but on a structured system of indicators. The use of ROH- and HBD-based information in genomic mating schemes makes it possible to simultaneously maintain genetic progress while limiting the rate of accumulation of recent inbreeding [53]. Thus, genomic inbreeding becomes not only a diagnostic parameter but also a controllable variable within the breeding process.

The available evidence suggests a conceptual model where inbreeding is viewed as a multilayered structure of autozygosity. This structure consists of an ancient background that has been partially purged of deleterious alleles. In contrast, more recent segments carry the primary inbreeding load. The biological consequences are determined by their relative proportions, their distribution across the genome, and their interaction with the direction of selection [54,55]. Such an interpretation integrates the classical theory of inbreeding with modern genomic approaches into a unified methodological framework.

Methodological Limitations and Reproducibility Requirements for ROH and HBD Analyses.

Despite the widespread use of ROH- and HBD-based approaches, the correctness of their application depends on several analytical decisions that can substantially influence the interpretation of results.

1. Marker density and quality.

Medium-density SNP panels limit the detection of short ROH and may bias estimates toward recent inbreeding. When analyzing populations with deep demographic histories, the potential underestimation of ancient autozygosity should therefore be taken into account.

2. ROH detection parameters.

The minimum segment length, and the number of SNPs, and the allowed number of heterozygous and missing calls should be justified based on the characteristics of the dataset. Universal thresholds do not exist; parameters should be selected according to marker density and expected error rates.

3. Genome assembly and recombination scale.

The interpretation of ROH length as an indicator of inbreeding age is valid only when an appropriate recombination map is used. Analyses expressed in megabases (Mb) without accounting for recombination rate variation may lead to oversimplified interpretations of temporal structure.

4. Choice of HBD model and number of classes.

In probabilistic models, excessive subdivision of age classes may lead to overfitting, whereas overly coarse stratification may obscure differences between ancient and recent layers. The optimal number of classes should therefore be determined based on sample size and the demographic characteristics of the population.

5. Interpretation of local ROH segments.

The presence of “ROH islands” does not necessarily indicate negative effects; such regions may reflect signatures of selection or historical population bottlenecks. Consequently, functional annotation and comparison with quantitative trait loci (QTL) are essential components of the analysis.

6. Cross-study comparability.

To ensure reproducibility, studies should report in detail the filtering parameters, the analyzed genome length, the genome assembly used, as well as the software and package versions applied. Without such information, cross-population comparisons lose methodological reliability.

## 3. Conclusions

Pedigree-based and genomic inbreeding coefficients reflect overlapping but not fully equivalent aspects of autozygosity; the discrepancies between them have both biological and methodological origins. Approaches based on ROH and HBD provide a temporal interpretation of inbreeding, but they are sensitive to marker density and analytical parameters. A universal inbreeding coefficient does not exist; the choice of method should be determined by the specific objective—whether monitoring short-term dynamics, reconstructing demographic history, or managing genetic diversity.

Standardization of analytical protocols and transparency in reporting are essential prerequisites for ensuring the comparability of results across studies and populations. The integration of genomic inbreeding metrics into optimal contribution strategies and diversity management frameworks is therefore a necessary condition for achieving sustainable genetic progress in modern livestock breeding.

## Figures and Tables

**Table 1:** Comparative characteristics of the PLINK, DetectRUNS, and RZooRoH methods.

Method	Approach	Main Principle	Advantages	Limitations
PLINK	Observational (sliding window)	Detects ROH based on consecutive homozygous SNPs using a sliding window across the genome	High computational efficiency; fast processing of large datasets; low memory requirements; flexible parameter settings; widely used and well documented in the literature	Highly sensitive to parameter choice; lack of universal thresholds; limited modeling of demographic history; does not estimate the age or origin of ROH; risk of false-positive and false-negative ROH detection
DetectRUNS	Observational/deterministic algorithms	Identifies ROH using several algorithms (sliding window, consecutive homozygous SNPs, combined method) with extended visualization tools	Rich visualization capabilities; detailed classification of ROH by length; useful for distinguishing recent and ancient inbreeding; built-in tools for statistical analysis; integration with the R ecosystem	Higher computational requirements than PLINK; slower with high-density SNP data; requires R programming skills; deterministic detection approach
RZooRoH	Model-based (Hidden Markov Model, HMM)	Models the genome as a mosaic of HBD and non-HBD segments and classifies ROH into HBD classes corresponding to different ancestral generations	Probabilistic framework; accounts for uncertainty in ROH detection; robust to genotyping errors and missing data; incorporates demographic history; distinguishes recent and ancient inbreeding; high accuracy	Computationally intensive; longer runtime for large datasets; requires expertise in HMM and advanced statistical interpretation; complex model parameterization (choice of HBD classes and HMM parameters)

**Table 2:** Practical interpretation of inbreeding coefficients across different analytical levels.

Coefficient	Main Level of Information	Most Informative for
F_ped	Pedigree/expected autozygosity	Monitoring pedigree structure, long-term mating management
F_GRM	Marker-based genomic similarity	Genomic relationship structure, selection models
F_ROH	Segment-based realized autozygosity	Recent inbreeding, ROH burden, inbreeding depression
F_HBD	Probabilistic age-partitioned autozygosity	Temporal stratification of inbreeding, ancient vs. recent components

## Data Availability

The original contributions presented in this study are included in the article. Further inquiries can be directed to the corresponding authors.
